# Enhancing Discharge Performance and Image Lag Characteristics in PIN Diode X-Ray Sensors with a Reset Transistor

**DOI:** 10.3390/s26030929

**Published:** 2026-02-01

**Authors:** Hanbin Jang, Jinwook Heo, Moonjeong Bok, Eunju Lim

**Affiliations:** 1 Department of Convergent System Engineering, Dankook University, Yongin 16890, Republic of Korea; lloyd.jang@dankook.ac.kr (H.J.);; 2 duoPIX-ray Inc., Yongin 16914, Republic of Korea

**Keywords:** X-ray image sensor, image lag, thin film transistor, PIN diode, InGaZnO

## Abstract

With the advent of electric vehicles, the demand for non-destructive inspection methods for battery evaluation has increased. Among various requirements, achieving high-frame-rate performance is particularly critical for rapid inspection in end-user systems. However, image delay, which increases with frame rate, has emerged as a significant challenge due to inherent limitations in sensor design. As a result, extensive research has been conducted to improve image lag performance. In this study, we conducted an in-depth analysis of the fundamental causes of image lag in image sensors. Based on these findings, we fabricated a novel sensor with a reset transistor separate from the readout transistor used for data transfer. This approach effectively increased the reset current of the photodiode, significantly reducing image lag. The transistor material used in this study was InGaZnO, which showed a significant improvement in image lag compared to conventional methods. By introducing a dedicated reset transistor, the allowable reset current of the PIN diode was increased by a factor of 100 compared to the ROIC-limited condition, resulting in a significant reduction in image lag from 3.8% (STS) to 0.9% (DTS) under high-frame-rate operation. This research provides a theoretical basis for proposing various new X-ray digital image sensor structures.

## 1. Introduction

With the rapid growth of electric vehicle technologies, non-destructive inspection methods for lithium-ion batteries have attracted significant attention, particularly for in situ and high-speed evaluation of internal structures [1,2]. In response to these market conditions, indium gallium zinc oxide (IGZO) thin-film transistors have been actively investigated for flat-panel X-ray detectors due to their higher carrier mobility, lower noise characteristics, and improved readout speed compared to conventional a-Si TFTs [3,4]. IGZO-based flat-panel detectors have demonstrated competitive imaging performance in terms of MTF, NPS, and DQE, enabling their application in both 2D and 3D X-ray imaging systems [3]. However, despite these advantages, as the frame rate increases, image lag has emerged as a critical challenge in flat-panel X-ray detectors due to inherent limitations in pixel-level charge reset mechanisms and readout circuitry [5]. Insufficient discharge of residual charge accumulated in the photodiode during successive exposures can lead to temporal signal overlap between frames, resulting in image degradation. Consequently, extensive research efforts have been devoted to improving image lag performance through advances in pixel architecture, readout schemes, and detector operating conditions. The image lag can be attributed to the residual charge that accumulates in the depletion region of the P-Type/Intrinsic/N-Type(PIN) diode within the X-ray sensor. This results in an inadequate discharge of the energy level formed inside the PIN diode through the X-ray irradiation, which affects the subsequent irradiation [6]. The lag results in a blurring of the final image and a reduction in its effectiveness as a data source [7]. In order to address this issue, a methodology for equalizing the level of the PIN diode utilizing an electroluminescent sheet, which is a physical light-emitting material, was previously proposed [8]. However, this method requires a high current, which presents a challenge in terms of practical implementation. Furthermore, the acquisition of an empty image for diode resetting between images may prove advantageous; however, this has the disadvantage of reducing the speed of dynamic detectors [7]. Software alternatives for image lag include methods that use various recursive models [9,10,11,12]. However, any software-related method requires a significant amount of data to process images without artefacts or a separate processor to improve speed performance.

Furthermore, the afterglow represents another factor that causes image lag. The primary scintillator materials employed are terbium-doped gadolinium oxysulfide (Gd_2_O_2_S:Tb) and thallium-doped cesium iodide (CsI:Tl) [13,14,15]. In comparison to other scintillator materials, these possess a greater capacity for large-area utilization, which is a significant advantage. This is attributable to the simplicity of their processing and the high efficiency with which they convert light. CsI:Tl scintillators typically exhibit a light yield in the range of approximately 50,000–65,000 photons/MeV, depending on the fabrication method, dopant concentration, and measurement conditions [13,16]. It is important to note, however, that all scintillators exhibit an afterglow effect, which is contingent upon the excitation characteristics of the material in question. Consequently, there has been a concerted effort by several scholars to enhance this effect [17]. The CsI layer is typically grown at a slow rate using an evaporation technique. However, this process is inherently challenging, and even slight alterations in the process conditions can result in significant changes to the columnar structure, which ultimately impairs the final light transmission efficiency [18,19,20,21]. The enhancement of image lag through the modification or improvement of scintillator materials is a complex undertaking, facing numerous challenges.

In addition to material-dependent afterglow effects, several studies have reported temporal delay reduction using pulsed X-ray sources with extremely short exposure durations and high peak power. Such approaches, which increase the ratio of reset or readout time relative to exposure time, have been shown to suppress residual signal components in industrial scintillators. These studies highlight alternative system-level strategies for mitigating temporal delays in high-speed X-ray imaging [22,23,24].

In order to eliminate lag without resorting to a reduction in speed or the introduction of side effects, it is necessary to examine the structural causes of this phenomenon in conventional X-ray sensors. In a sensor, a PIN diode with a high resistance component is paired with a thin-film transistor (TFT) in a unit pixel. A single TFT sensor (STS) array is used in a conventional X-ray digital image sensor, with one TFT per unit pixel [25,26,27,28]. The TFT is linked to a readout IC (ROIC) via a data electrode, enabling control of the discharge (reset) of the PIN diode and perform readout through the reference voltage of the ROIC. However, the ROIC requires a relatively low-current reference voltage to limit heat generation and noise. Consequently, in the existing structure, the amount of discharge per unit time is reduced due to an insufficient reset time in the high-speed readout condition. This results in the accumulation of charge leftovers, which ultimately leads to an image lag [29,30].

The objective of this study was to analyze the structural and electrical factors responsible for the image lag in the high-speed X-ray digital image sensors. In order to improve the image lag, a reset transistor inducing a high current was added to the conventional photon-integrating X-ray digital image sensor. A dual TFT sensor (DTS), which is a two-dimensional array sensor, was implemented to enhanced the image lag in comparison to the STS. Furthermore, we conducted a comparative analysis of the sensitivity performance that may result from alterations in the X-ray sensor structure. Through simulation, we also verified electrode heating due to overcurrent. This study offers a valuable opportunity to present diverse perspectives in the field of X-ray sensors, which have not been structurally or performance-differentiated for an extended period.

## 2. Experimental Detail

### 2.1. Sensor Design

**Figure 1:**
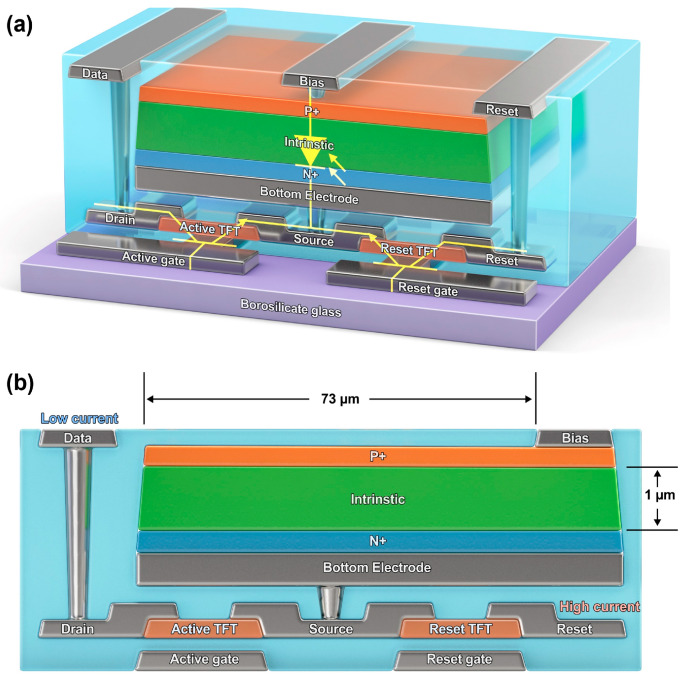
(**a**) Schematic and (**b**) Cross-Section of the Designed DTS.

To implement the high-current reset environment of the PIN diode, arranging separate reset TFT and electrodes in a conventional sensor structure is essential, as indicated in Figure 1a. A layout editor was used for designing, and Figure 1b represents the cross-sectional view of the newly designed sensor. InGaZnO (IGZO) thin films were formed on the same layer as readout and reset TFTs, within a unit pixel of 73 μm. The fabricated sensor consists of a 2048 × 2048 pixel array with a pixel pitch of 73 μm, corresponding to a full-area flat-panel detector configuration. The Width/Length (W/L) of the TFT was set to 20/5 μm. Each TFT was connected to a source electrode composed of one Molybdenum (Mo)/Aluminum (Al)/Mo. After forming a via hole in the passivation layer that protected the source electrode, it was connected to the lower electrode of the PIN diode through the Mo/Al/Mo contact. Each drain electrode in direct contact with the TFT was configured, and that connected to the readout TFT was connected to the data electrode.

Thus, the drain electrode was connected to the ROIC through a via hole. The drain electrode connected to the reset TFT was used as the reset electrode. A PIN diode was formed as the upper layer of the TFT, and the thickness of the intrinsic layer of the PIN diode was 1.0 μm. Thus, the fabricated sensor had a reset TFT added to the conventional sensor with only a readout TFT. In the fabricated sensor, the source electrodes of the readout and reset TFTs were directly connected to the bottom electrode of the PIN diode.

In addition, the experimental results of conventional and improved structures were evaluated and derived using a single sensor. The electrical characteristics of semiconductor devices may differ significantly depending on their microscopic structure, and some performance deviations can occur depending on the date of production [31]. Therefore, additional electrodes and reset transistors were placed under the lower electrode of the PIN diode. In this case, the same environment as that of the existing STS can be maintained by simply supplying an off-voltage to the reset TFT.

The scintillator layer was fabricated by vacuum evaporation of a CsI:Tl mixture. The maximum deposition temperature was maintained at 180 °C, resulting in a final scintillator thickness of approximately 300 µm. Immediately after CsI deposition, the scintillator was insulated by laminating a white PET protective layer.

### 2.2. Driving Sequence and Electrical Parameters

**Figure 2:**
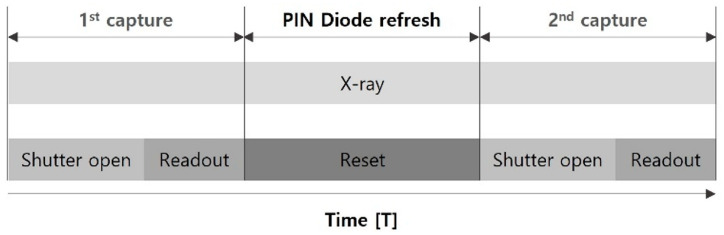
X-ray Image Sensor Driving Sequence.

The electrical conditions for the image acquisition of the manufactured sensor were defined, and the driving sequence was simulated. The Reset was used to reset the transistor; the scintillator converted the energy of X-ray to visible light in the PIN diode (shutter open); and the final accumulated charge was read by the integrator Read (Figure 2). To compare the performances of DTS and STS, STS fixed the reset TFT to an off state and performed a reset with the readout TFT for the reset time (Table 1).

Additionally, the electrical conditions for driving the STS and DTS modes were defined. The reset current in the STS was set to 40 μA as the allowable current of the charge amp of the ROIC; therefore, a DC power supply with a maximum current specification of 2 A was used for a DTS with a higher current environment than ROIC. The on and off voltages of all transistors were set to 18 and −8 V, respectively. The bias voltage for the PIN diode was set within the range of −3 to 3 V. However, the reset transistor of the STS was set to off state continually. The reference voltage during the readout and reset was 1.5 V in accordance with the ROIC specifications. The voltage of the drain electrode connected to the reset transistor for DTS operation was identical to the reference voltage of the ROIC, namely 1.5 V.

**Figure 3:**
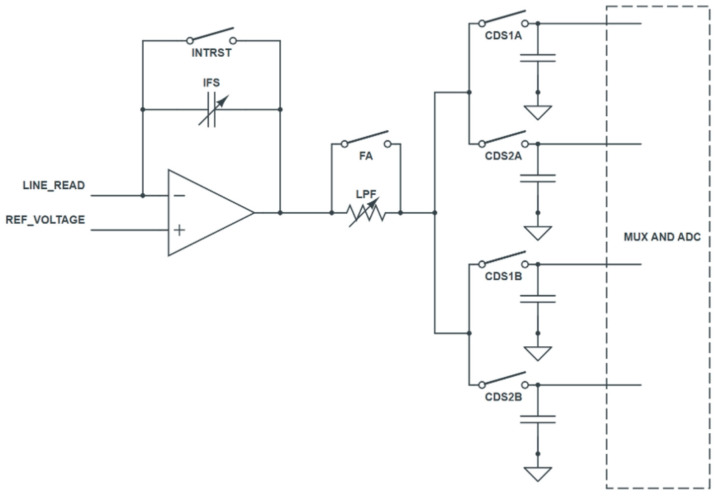
Simplified Block Diagram of the ROIC.

**Figure 4:**
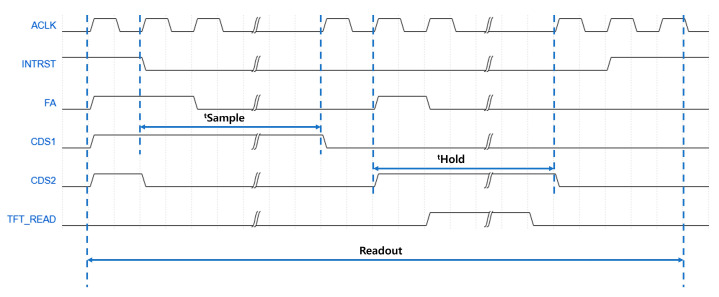
Sequence of the Readout Section.

In this study, ADI AD71182 was used as the readout integrated circuit (ROIC). A simplified block diagram of the IC is shown in Figure 3, and The detailed readout sequence of the readout section defined in Figure 2 is illustrated in Figure 4. The frame rate was adjusted by setting tSample and tHold to 20 μs for 30 fps and 60 μs for 10 fps, respectively, while maintaining the same ratio between them. The time constant of the low-pass filter (LPF) was set to 1.3 μs, and the integrator gain was determined by an integration capacitance of 1 pF. To minimize the effect of TFT switching noise during the hold period, both the TFT turn-on and turn-off operations were included within the hold section. The TFT was turned off at least 3 μs before the end of the hold period to ensure signal stability.

### 2.3. Device Characteristics Measuring

Before measuring the image performance, the unit element characteristics of the X-ray image sensor were verified to determine its I-V characteristics. All the measurements were performed at room temperature. The voltage applied to the TFT (Vds 0.1 V) was in the range of −20 to 20 V. The current at the data electrode, corresponding to each voltage, was measured. The bias voltage for PIN diode was set in the range of −3 to 3 V, and the current at the lower electrode of the PIN diode was recorded. Since 100 PIN diodes were connected in parallel during the measurements, all the data were recorded in hundredths.

### 2.4. Joule Heating Effect Simulation

The Joule heating effect was simulated to determine the heat generation corresponding to an increase in current, and COMSOL Multiphysics was used as the simulator. The electrode was made of aluminum alloy with a width and thickness of 7 μm and 8000 Å, respectively, and 2 μm of the upper part of electrode was covered with silicon nitride. The length of the conductor was set to 73 μm (identical to the unit pixel size). The ambient temperature was set to 20 °C. The values provided by the COMSOL library were used as material parameters. To calculate heat transfer in the solids, the formula provided by the simulation tool was used. Simulations were conducted at a pressure of 1 atm. In terms of current conditions, assuming a maximum current of ROIC of 40 μA is consumed by 1024 pixels, the maximum current consumed by a unit pixel is approximately 0.039 μA. Thus, 0.039 μA was used as a reference model, and a condition of 100 times higher current (3.9 μA) was also simulated.

## 3. Results and Discussions

**Figure 5:**
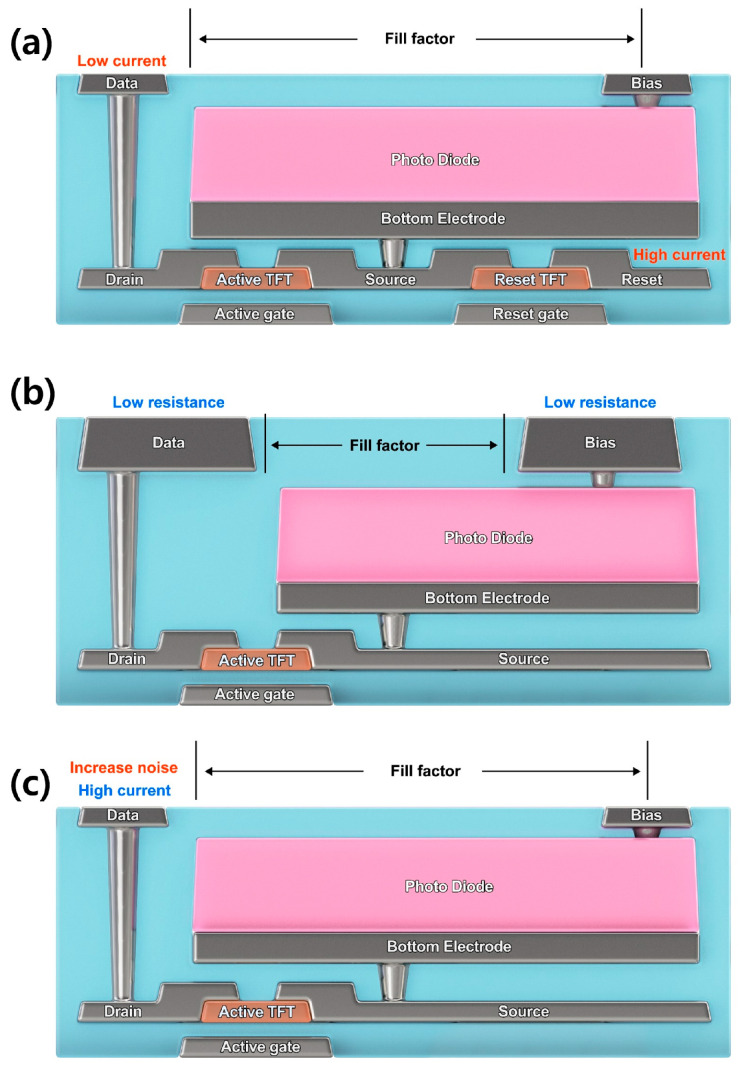
Considered Design Method to Improve Image Lag, (**a**) DTS Image Sensor; (**b**) Decrease in Data Resistance; (**c**) Use of High-Current ROIC.

In general, increasing the surface area of each electrode is essential for reducing its resistance. However, an increase in the ratio of the surface area of the electrode per unit pixel results in a loss of fill factor, as shown in Figure 5b. Alternatively, the drain current can be improved without loss of the fill factor by applying an ROIC, which allows for a high current, as shown in Figure 5c. However, the use of high voltage inside the ROIC results in increased heat generation and noise. Accordingly, the placement of the TFT below the PIN diode, as adopted in this study, represents an effective method for increasing the reset current without compromising the fill factor or introducing electrical noise (Figure 5a).

**Figure 6:**
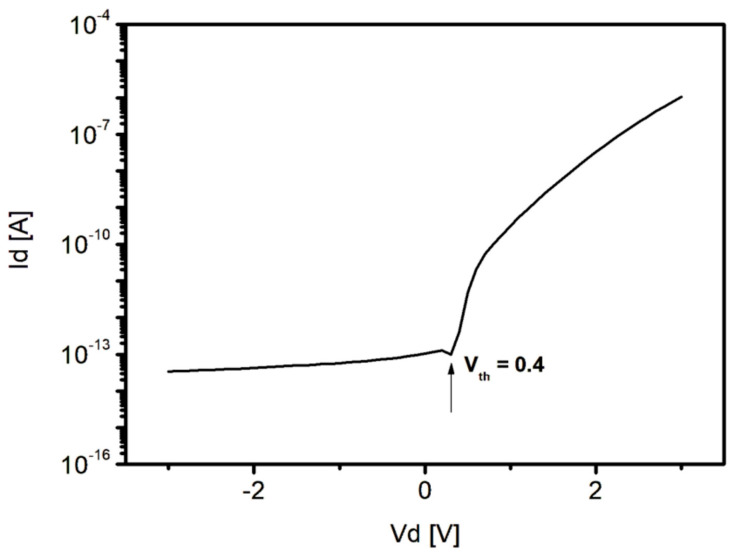
I-V Characteristic of PIN Diode on the Fabricated Sensor. The Arrow Indicates V_th_.

**Figure 7:**
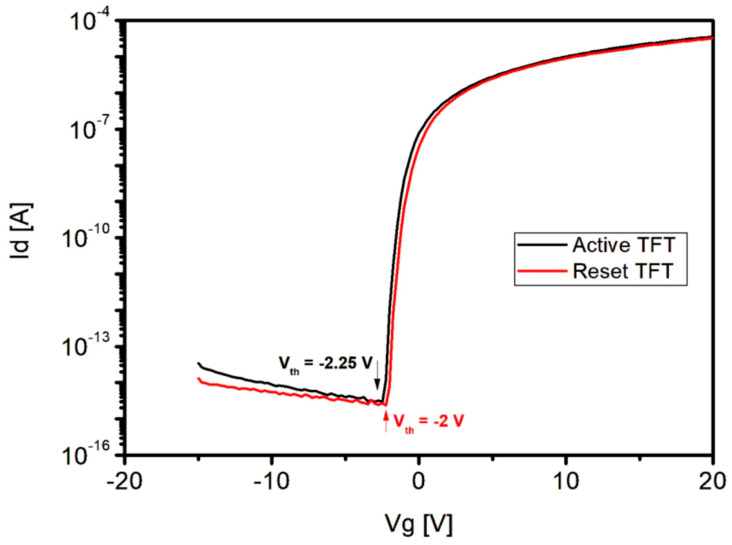
I-V Characteristics of Readout and Reset TFTs on the Fabricated Sensor. The Arrow Indicates V_th_.

Despite the structural transformation of the sensor, the threshold voltage (Vth) of the PIN diode was 0.4 V (Figure 6) and that of the reset TFT was −2.0 V, at a level similar to −2.25 V of readout TFT. For both the readout and reset TFTs, the stable off and on state was achieved at approximately −5 and 15 V (Figure 7), respectively. In particular, Readout and reset TFTs were formed together in the same layer, and no degradation in the TFTs performance was observed. The PIN diode was also turned on/off normally.

**Figure 8:**
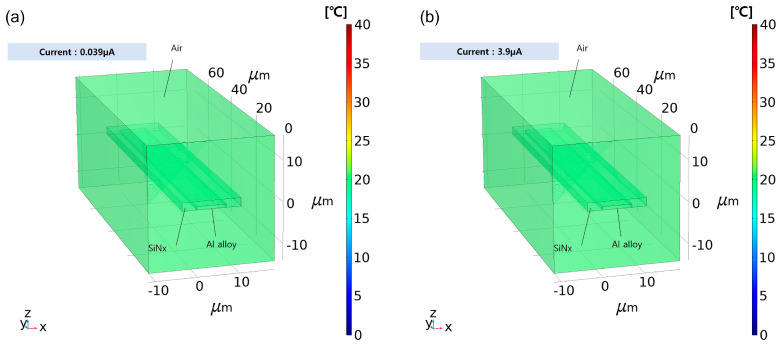
Simulation of Joule Heating Effect of Electrode with Different Values of Current: (**a**) 0.039 μA (the Maximum ROIC Current), and (**b**) 3.9 μA (100× of the ROIC Current).

An increase in current immediately generates heat due to the resistive components of the electrode [32]. To this end, the increase in current and amount of heat generation were confirmed through numerical simulation using the Heat Transfer in Solids module of COMSOL Multiphysics. Figure 8 shows a graph visualizing the heat generation simulation results on the electrode in the sensor: Figure 8a, b indicate the result when reset by the maximum current allowed by the ROIC, as in the conventional method and when the current is 100 times higher than the ROIC maximum current, respectively. In Equation (1), *k* refers to thermal conductivity, *ρ* refers to density, and *C_p_* refers to heat capacity at constant pressure. Each harsh current condition, compared to actual sensor operating environments with line times of less than tens of microseconds, was continued for 1 s; at 0.039 μA, the temperature of the overall model was confirmed to be 20 °C (the ambient temperature). Even at 3.9 μA, which was 100 times higher than reference, the temperature at the center of the electrode was confirmed to be 20.001 °C, which was only 0.001 degrees higher than the ambient temperature. Therefore, according to the simulation results obtained using COMSOL Multiphysics and the heat generation formula provided by the software, the heat generation was negligible.

(1) $$\rho C_{p}\frac{\delta T}{\delta t}+\rho C_{p}u\nabla T+\nabla q=Q+Q_{ted},q=-k\nabla T$$

**Figure 9:**
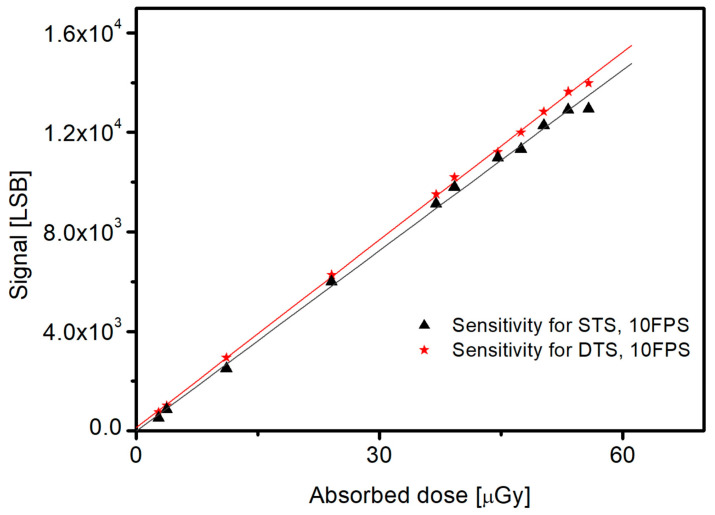
Sensitivity Performance for DTS and STS.

Another important performance indicator of X-ray detectors is sensitivity [33]. The sensitivity was defined as the slope of the linear fit between the digital output signal level (LSB) and the absorbed X-ray dose. The absorbed dose for each measurement was experimentally determined using a calibrated dosimeter. During the measurements, the tube voltage was fixed at 73 kVp, while the tube current–exposure time product (mAs) was varied to control the dose. For each exposure condition, the average signal level obtained under X-ray irradiation was divided by the corresponding measured absorbed dose, and the resulting data were fitted linearly to extract the sensitivity in units of LSB per unit dose. Consequently, the sensitivity performance of 241 and 258 for STS and DTS, respectively, suggested no specific performance differences (Figure 9). This result indicates that increase in the reset current of the PIN diode does not contribute to improving sensitivity. To increase the sensitivity, a direct improvement of the PIN diode, such as increasing the fill factor, is required.

**Figure 10:**
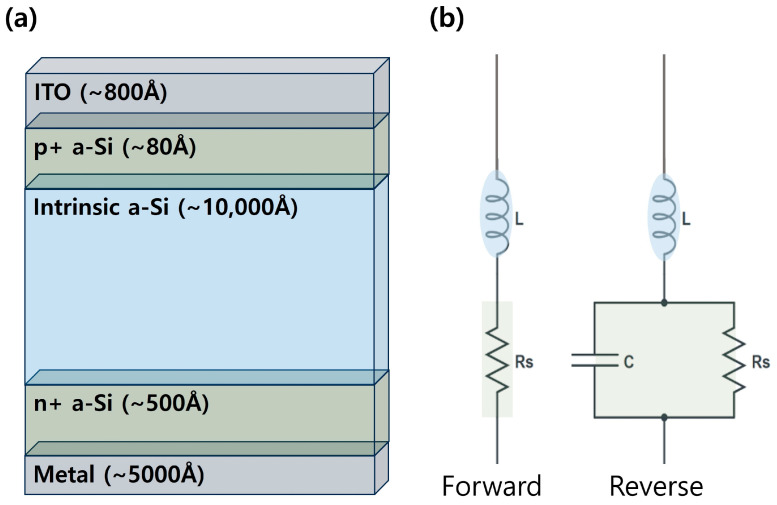
a-Si PIN Diode Structure and Equivalent Circuit for (**a**) Forward and (**b**) Reverse States.

The feasibility of using a high current in the PIN diode to improve the lag performance should be considered theoretically to understand the mechanism of image lag improvement. A typical PIN diode is shown in Figure 10a where n+, intrinsic, and p+ a-Si are arranged in that order with Al or Cr as the lower electrode. Indium tin oxide (ITO) with high transmittance is generally used as the upper electrode considering its light reception efficiency [34]. When light is absorbed by the PIN diode, the electron–hole pairs within the intrinsic layer separate owing to the electric field, and the band gap functions as energy [35]. With this background, the equivalent circuit, reflecting the characteristics of each bias state in these PIN diodes, can be expressed as Figure 10b. The PIN diode is multilayered, and the inductance mainly originates from the “I” (intrinsic or neutral layer) between the “P” (positive) and “N” (negative) layers. Although capacitance and resistance are mainly generated at P- and N-intrinsic surfaces, they may also appear at various locations within the multilayer structure of a PIN diode [36]. However, the resistance (*Rs*), specifically in the forward-bias state, on resetting the PIN diode is significant [37,38,39]. According to previous studies, the amount of charge (*Q*) of the PIN diode in the forward bias is proportional to the forward bias current (*I_F_*) and carrier lifetime (*τ*) (Equation (2)). The i-region resistance *R_S_* is directly proportional to i-region width (*W*) and inversely proportional to the sum of electron mobility (*µ_N_*) and hole mobility (*µ_P_*) multiplied by *Q*. Therefore, *Rs* is responsible for the amount of discharge per unit time, which can be improved by increasing the current (*I_F_*) (Equation (3)) [37,38,39].

(2) $$Q=I_{F}\tau$$

(3) $$R_{S}=\frac{W^{2}}{\left(\mu_{N}+\mu_{p}\right)Q}$$

Thus, the forward bias current (*I_F_*) was increased using a separate reset transistor added in this study. Consequently, the discharge rate of the PIN diode improved proportionally, effectively reducing the residual charge of the PIN diode. This ultimately improved the image lag.

The image lag was evaluated according to the image delay performance measurement method presented in IEC62220-1 [40,41,42], and the performance was evaluated under 10 and 30 frames per second (FPS) conditions of STS and DTS, respectively. The difference in performance was confirmed based on the frame rate. 7252X (Hamamatsu Photonics K.K., Shizuoka, Japan)was used as an X-ray device for generating the image lag, with X-ray energy conditions of 70 kV and 50 mA, and a source-to-image receptor distance (SID) of 1500 mm.

**Figure 11:**
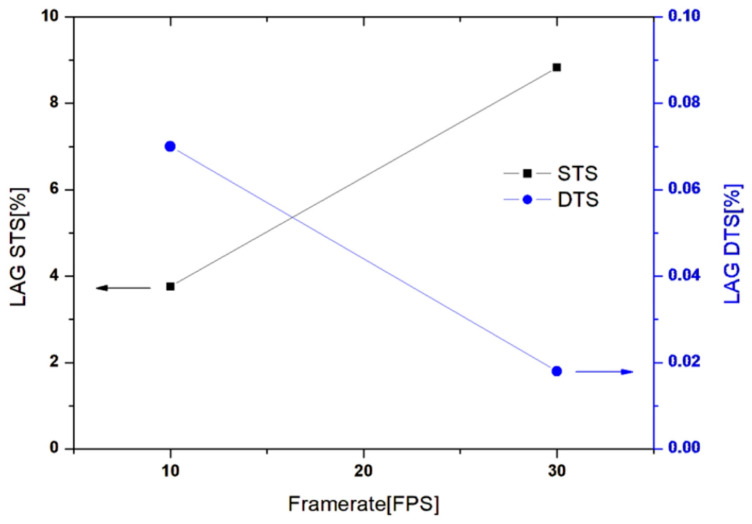
Image Lag Test Results for STS and DTS.

The test results confirmed that as the frame rate increased from 10 to 30 FPS, the lag increased and decreased in STS and DTS by approximately 2.3 and 4.1 times, respectively. A significant improvement in the degree of occurrence of image lag in DTS compared with STS in actual images was observed with the naked eye (Figure 11). Notably, the observed decrease in image lag with increasing frame rate in the DTS configuration can be plausibly attributed to the enhanced reset efficiency relative to the accumulated offset charge in the PIN diode. As the frame rate increases, the exposure time is reduced, leading to a lower amount of offset charge accumulated during each frame, while the reset current provided by the DTS remains sufficient to effectively remove the residual charge. In contrast, in the STS configuration, the reset process is limited by the ROIC, and the reduced frame time at higher frame rates may result in insufficient charge removal, causing the image lag to remain constant or slightly increase. Although this explanation is consistent with the observed trends, a detailed quantitative analysis of this mechanism is beyond the scope of the present study.

**Figure 12:**
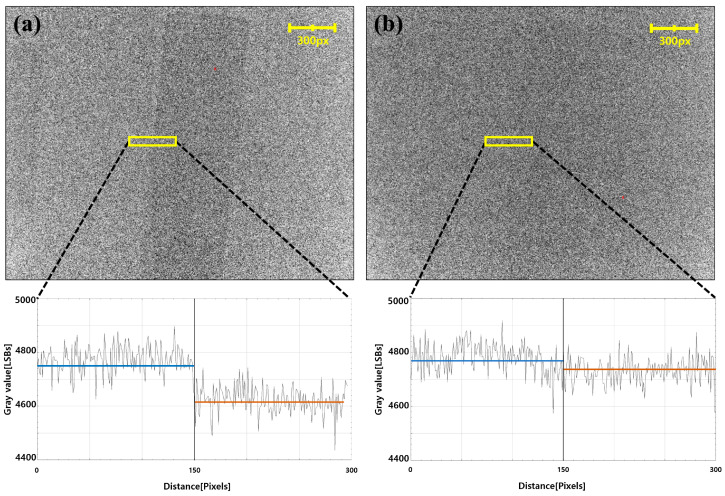
Image Lag of the 18650 Batteries, and the Gray Value Profile from a Distance: (**a**) STS and (**b**) DTS. The Dotted Line Indicates the Profiled Area. The blue line indicates the average signal of the air region, and the orange line indicates the average signal of the 18650 battery region.

After acquiring the 18650 battery image at 70 kV and 25 mA, a dark frame (an image after the X-rays were completely off) was obtained. Subsequently, the areas expecting image lag of the battery and air were profiled, and the average of each area was confirmed. As a result, STS exhibited a deviation of approximately 164LSB (3.8%) owing to image lag (Figure 12a); however, DTS exhibited a clear decrease in lag compared to STS, with 44LSB (0.9%), which is close to 0% (Figure 12b). Thus, we experimentally confirmed that increase in the reset current of the PIN diode has a direct and practical effect on reducing lag.

## 4. Conclusions

In this study, a dual-transistor structure (DTS) was proposed to effectively reduce image lag in IGZO-TFT-based flat-panel detectors by enhancing the reset capability of the PIN diode. By decoupling the reset current limitation imposed by the ROIC, the proposed structure enables a significantly higher reset current, leading to a substantial reduction in residual charge and image lag, particularly under high-frame-rate operating conditions. Experimental results demonstrated that the image lag was reduced from 3.8% in the single-transistor structure (STS) to 0.9% in the DTS configuration, confirming the effectiveness of the proposed approach.

While this work primarily focuses on image lag reduction through an enhanced detector-level reset mechanism, other image quality metrics such as modulation transfer function (MTF), noise power spectrum (NPS), and detective quantum efficiency (DQE) were not explicitly evaluated and remain important topics for future investigation. In addition, variations in receiver geometry, pixel configuration, and array design may influence the effective detection area and inter-pixel interactions, which should be carefully considered in future large-area detector implementations.

Although image lag can also be affected by factors such as scintillator afterglow or X-ray source characteristics, these effects were not addressed in the present study. Instead, this work focuses on detector-level lag reduction by improving the reset process of the PIN diode, independent of scintillator properties or exposure waveforms. Importantly, the proposed DTS is not limited to the specific ROIC used in this study and can be readily applied to other ROIC architectures that impose similar reset current limitations. As such, the DTS provides a practical, scalable, and broadly applicable solution for high-speed X-ray imaging systems requiring low image lag without increasing overall system complexity.

## Figures and Tables

**Table 1:** Transistor Activation Status for Each Sequence Step.

Mode	Gate	Reset	Shutter Open	Readout
STS	Active Gate	On	Off	On
	Reset Gate	Off	Off	Off
DTS	Active Gate	Off	Off	On
	Reset Gate	On	Off	Off

## Data Availability

The original contributions presented in this study are included in the article. Further inquiries can be directed to the corresponding author(s).
